# Hyperacusis in the Adult *Fmr1*-KO Mouse Model of Fragile X Syndrome: The Therapeutic Relevance of Cochlear Alterations and BKCa Channels

**DOI:** 10.3390/ijms241411863

**Published:** 2023-07-24

**Authors:** Celeste Ferraguto, Yohan Bouleau, Thibault Peineau, Didier Dulon, Susanna Pietropaolo

**Affiliations:** 1 Univ. Bordeaux, CNRS, EPHE, INCIA, UMR 5287, F-33000 Bordeaux, France; 2 Neurophysiologie de la Synapse Auditive, Université de Bordeaux, INSERM UA06, F-33000 Bordeaux, France; 3 Institut de l’Audition, Centre Institut Pasteur, Inserm UA06, F-75012 Paris, France

**Keywords:** neurodevelopmental disorders, potassium channels, hearing loss, startle reflex, auditory brain stem responses

## Abstract

Hyperacusis, i.e., an increased sensitivity to sounds, is described in several neurodevelopmental disorders (NDDs), including Fragile X Syndrome (FXS). The mechanisms underlying hyperacusis in FXS are still largely unknown and effective therapies are lacking. Big conductance calcium-activated potassium (BKCa) channels were proposed as a therapeutic target to treat several behavioral disturbances in FXS preclinical models, but their role in mediating their auditory alterations was not specifically addressed. Furthermore, studies on the acoustic phenotypes of FXS animal models mostly focused on central rather than peripheral auditory pathways. Here, we provided an extensive characterization of the peripheral auditory phenotype of the *Fmr1*-knockout (KO) mouse model of FXS at adulthood. We also assessed whether the acute administration of Chlorzoxazone, a BKCa agonist, could rescue the auditory abnormalities of adult mutant mice. *Fmr1*-KO mice both at 3 and 6 months showed a hyperacusis-like startle phenotype with paradoxically reduced auditory brainstem responses associated with a loss of ribbon synapses in the inner hair cells (IHCs) compared to their wild-type (WT) littermates. BKCa expression was markedly reduced in the IHCs of KOs compared to WT mice, but only at 6 months, when Chlorzoxazone rescued mutant auditory dysfunction. Our findings highlight the age-dependent and progressive contribution of peripheral mechanisms and BKCa channels to adult hyperacusis in FXS, suggesting a novel therapeutic target to treat auditory dysfunction in NDDs.

## 1. Introduction

Fragile X Syndrome (FXS) is a common inherited form of intellectual disability and the leading monogenic cause of autism spectrum disorder (ASD) [1]. FXS is a neurodevelopmental disorder (NDD) caused by a mutation in the X-linked *FMR1* gene leading to the absence of the FMRP protein, an RNA-binding protein that plays an important role in synaptic plasticity and neuronal maturation [2]. The lack of FMRP induces several neurobehavioral phenotypes in FXS patients including autistic symptoms, increased anxiety, hyperactivity, and sensory hyper-responsiveness [3,4,5]. In particular, auditory hypersensitivity or hyperacusis, i.e., an exaggerated perception of ordinary sounds, is often described in FXS patients [6,7] and may significantly contribute to their social and emotional abnormalities. Although hyperacusis represents a highly common and invalidating symptom of FXS and other NDDs [8,9,10], little is still known about its underlying mechanisms and specific pharmacological approaches are far from being identified. Existing treatments for FXS-linked hyperacusis include sound or cognitive behavioral therapies, and their efficacy is limited to helping patients cope with their auditory impairments in daily life. Identifying new pharmacological targets to develop effective treatments for hyperacusis is, therefore, an urgent need for FXS patients, which could have relevant implications for other NDDs.

Big conductance calcium-activated potassium (BKCa) channels represent promising candidates to treat auditory hypersensitivity in FXS. They are largely expressed throughout the auditory pathway and play a critical role in several aspects of auditory signaling [11,12]. In particular, a link between the loss of BKCa channels and progressive degeneration of the inner hair cell (IHC) ribbon synapses has been described in aging C57BL6/J wild-type (WT) mice and it was associated with a hyperacoustic peripheral phenotype [13]. Ribbon synapses are characterized by the presence of an electron-dense structure, i.e., the synaptic ribbon, which promotes rapid neurotransmitter release and synaptic transfer of auditory information from sensory IHCs to type I spiral ganglion neurons [14,15]. As BKCa channels regulate neurotransmitter release and neuronal triggering [16], they have already been proposed as emerging therapeutic targets for FXS [17,18,19] and other NDDs [18,20]. Reduced expression and functionality of these channels have been described in FXS patients and in several brain areas of FXS mice [17]. Hence, molecules activating BKCa channels were employed to treat FXS-like behavioral abnormalities in the preclinical model of FXS [17,21,22], the *Fmr1*-KO mouse line. This mouse model, lacking the FMRP protein, recapitulates most of the physical and neurobehavioral alterations of FXS patients [23,24] including hyperacusis [21,22,25,26]. These FXS-like phenotypic profiles were mostly investigated at adulthood (i.e., at 3 months of age or beyond), since this age seems the most suitable for detecting the most complete pathological behavioral phenotype in the *Fmr1*-KO mouse model [24]. Nonetheless, the effects of BKCa-based treatments on the adult acoustic phenotypes of *Fmr1*-KO mice have not been investigated in depth, as well as the role of potential alterations in the cochlear expression/functionality of BKCa channels in FXS mice.

The auditory phenotypes of *Fmr1*-KO mice were assessed in several previous studies, but often leading to discrepant results. Concerning the most common behavioral measures of acoustic functionality, a reduced acoustic startle response (ASR) was observed in *Fmr1*-KO mice in certain studies [27,28,29], while in others, the opposite mutant phenotype [21,22,30,31] was described. Concerning the electrophysiological evaluation of the acoustic phenotypes of *Fmr1*-KO mice, measures of auditory brainstem responses (ABRs) revealed increased or unaltered hearing thresholds and reduced wave I amplitudes, sometimes associated with alterations in other waves [32,33]. All these discrepancies could be explained by differences in the genetic background selected for implementing the *Fmr1* mutation, but also by the use of low versus high sound intensities to test ASR. The C57BL/6J (B6) background emerged as one of the most suitable to recapitulate FXS-like behavioral symptoms in the *Fmr1*-KO mouse model [34,35]. Furthermore, when ASR was assessed in *Fmr1* mutants of the B6 background using testing protocols based on low intensity stimuli (70–90 dB), a clear hyperacusis-like phenotype emerged in *Fmr1*-KO mice, similar to what described in FXS patients [22,30]. Although most studies employed adult mice, subtle differences in the age of testing could also have contributed to the inconsistent acoustic phenotypes previously reported in the *Fmr1* mouse model; this issue could be particularly relevant for the B6 background since this strain is characterized by early hearing loss, starting already at 6 months [36,37], i.e., an age largely used for the adult behavioral characterization of *Fmr1*-KO mice [24]. Furthermore, previous studies on the acoustic phenotypes of the *Fmr1*-KO mouse line investigated the underlying molecular mechanisms focusing on central auditory pathways, e.g., demonstrating an association between the hyperacusis displayed by *Fmr1* mutants and their impaired neuronal excitability in somato-sensory cortex, thalamic, and brainstem auditory nuclei [22,38,39,40,41,42,43]. Therefore, the contributions of peripheral pathways and molecular alterations in cochlear hair cells to the acoustic alterations of *Fmr1* mutant mice are still far from being elucidated.

Hence, in the present study, we first characterized in depth the peripheral acoustic phenotypes of the *Fmr1* mouse line at adulthood, combining behavioral (ASR), electrophysiological ABRs, and distortion product otoacoustic emissions (DPOAEs) in adult *Fmr1*-KO mice and their WT littermates. These functional analyses were combined with an immune-histochemical characterization of the cochlear expression of the main BKCa subunit, KCNMA1 [44], and the main component of ribbon synapses in the IHCs, RIBEYE [45]. Second, we investigated whether the peripheral acoustic phenotypes of adult *Fmr1*-KO mice could be rescued by the acute administration of a BKCa agonist, Chlorzoxazone (CHLOR). CHLOR, even following a single administration, was shown to rescue several behavioral abnormalities of *Fmr1* mutants, including their exaggerated acoustic startle response [21]. Here, we tested two separate batches of mice, i.e., at 3 and 6 months of age, in order to provide a more complete characterization of the adult auditory phenotype of the *Fmr1*-KO model, including two ages commonly used for the adult neurobehavioral assessment of this mouse line [24]. In all studies, WT littermate controls were included.

## 2. Results

### 2.1. Hyperacusis-like Phenotypes in Fmr1-KO Mice at 3 and 6 Months of Age: Behavioral and Electrophysiological Measurements

#### 2.1.1. Acoustic Startle Response (ASR)

**Figure 1:**
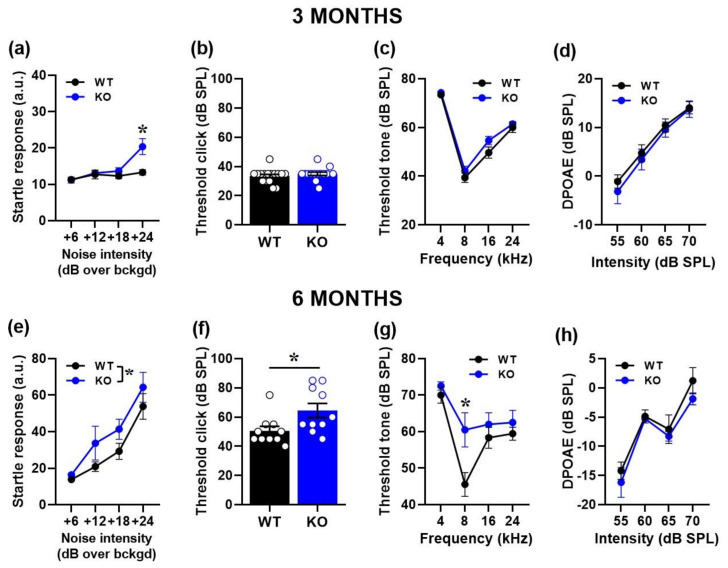
Behavioral and electrophysiological assessment of acoustic response in 3- and 6-month-old mice. Auditory responsiveness was first evaluated by the body startle over a background noise (bckgd) of 66 dB SPL (**a**,**e**). Hearing thresholds were then assessed by click- (**b**,**f**) and tone-ABRs (**c**,**g**) together with DPOAEs (**d**,**h**). Sample size: n = 16 (WT) and 14 (KO) for the 3-month-old group; n = 10 (WT) and 10 (KO) for the 6-month-old group. a.u. = arbitrary units. * *p* < 0.05. Data are mean ± SEM.

As expected, the ASR of all mice increased with the intensity of the acoustic stimulus at both ages [noise intensity effect at 3 and 6 months: F (1, 78) = 11.38, *p* < 0.0001 and F (1, 54) = 39.01, *p* < 0.0001, respectively]. *Fmr1*-KO mice showed an increased ASR compared to their WT littermates: while at 3 months, this effect was evident only at the highest intensity [interaction genotype x noise intensity: F (1, 78) = 3.58, *p* = 0.0176; Figure 1a], at 6 months, the hyperacusis of KO mice emerged at all intensities compared to WTs [genotype effect: F (1, 18) = 8.23, *p* = 0.012; Figure 1e].

#### 2.1.2. Auditory Brainstem Responses (ABRs) and Distortion Product Otoacoustic Emissions (DPOAEs)

At 3 months, no significant difference in the auditory threshold emerged between WT and KO mice either to the click [genotype effect: F (1, 28) = 0.87, ns; Figure 1b] or to the tone [genotype effect: F (1, 28) = 3.25, ns, its interaction with tone frequency: F (1, 84) = 0.61, ns; Figure 1c]. Mutant mice at this age also displayed unaltered DPOAEs [genotype effect: F (1, 28) = 0.24, ns; interaction genotype x noise intensity: F (1, 84) = 0.85, ns; Figure 1d].

At 6 months, hearing thresholds were significantly higher in KO mice compared to WT littermates: this genotype effect emerged in response to the click [genotype effect: F (1, 18) = 5.99, *p* = 0.025; Figure 1f], but also to the tone, when it was specific to the 8 kHz frequency [interaction genotype x noise frequency: F (1, 51) = 2.8, *p* = 0.049; Figure 1g]. DPOAEs did not also significantly differ between genotypes at this age [genotype effect: F (1, 18) = 0.79, ns; interaction genotype x noise intensity: F (1, 54) = 0.47, ns; Figure 1h].

**Figure 2:**
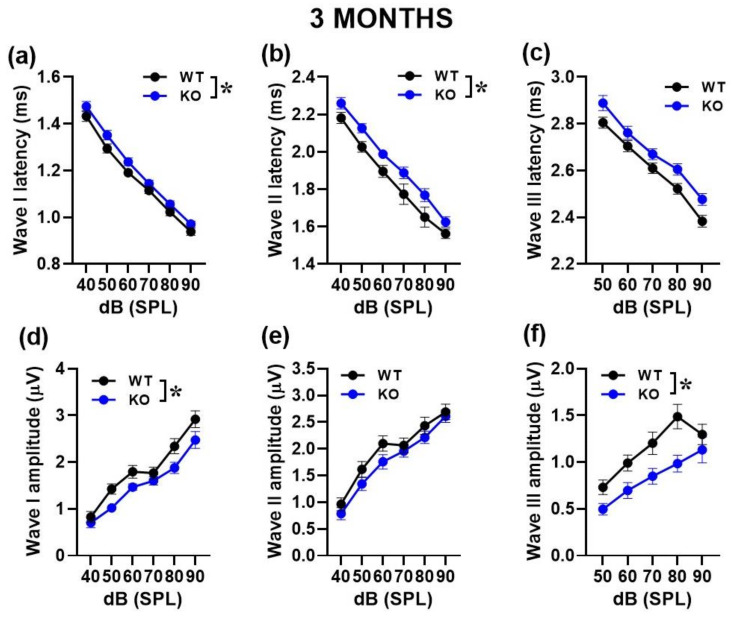
Quantification of latency and amplitude of ABR waves in 3-month-old mice. Latencies were calculated as the time when each peak occurred (**a**–**c**). Amplitudes were quantified as the voltage difference between the peak of the ABR and trough of the waveform for waves I–III (**d**–**f**). Sample size: n = 16 (WT) and 14 (KO). * *p* < 0.05. Data are mean ± SEM.

**Figure 3:**
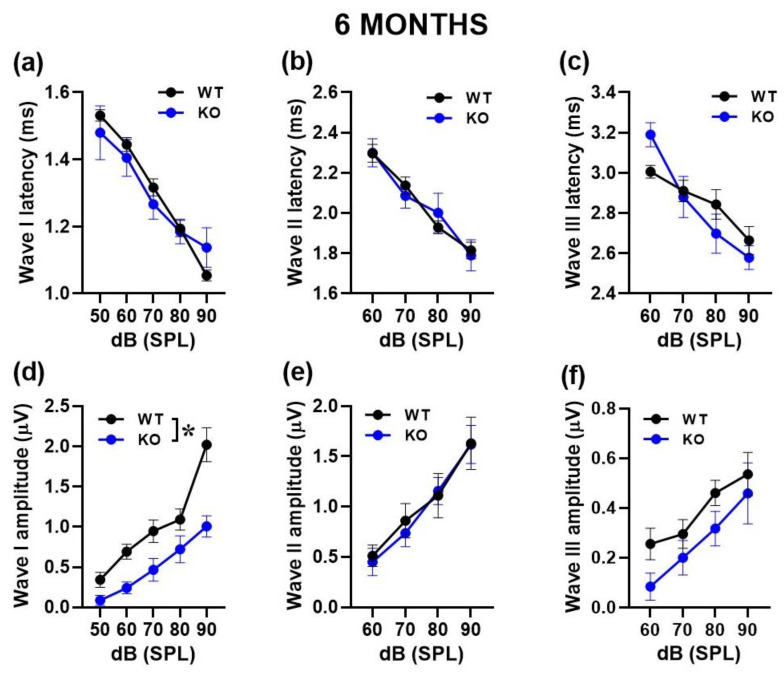
Quantification of latency and amplitude of ABR waves in 6-month-old mice. Latencies were calculated as the time when the height of each peak occurred (**a**–**c**). Amplitudes were quantified as the voltage between the peak of the ABR and trough of the waveform for waves I–III (**d**–**f**). Sample size: n = 10 (WT) and 10 (KO). * *p* < 0.05. Data are mean ± SEM.

We then performed a more detailed analysis of ABRs, comparing the latencies and amplitudes of the ABR wave-I, II and III as a function of sound intensity (Figure 2 and Figure 3). At both ages, the ABR waves were characterized by the typical decrease in peak latencies and increase in amplitudes with click intensity for wave I [noise intensity effect at 3 and 6 months for latency: F (1, 130) = 755.46, *p* < 0.0001 and F (1, 28) = 47.05, *p* < 0.0001; for amplitude: F (1, 140) = 109.47, *p* < 0.0001 and F (1, 72) = 64.77, *p* < 0.0001], for wave II [noise intensity effect at 3 and 6 months for latency: F (1, 130) = 125.72, *p* < 0.0001 and F (1, 33) = 99.75, *p* < 0.0001; for amplitude: F (1, 140) = 82.99, *p* < 0.0001 and F (1, 45) = 53.98, *p* < 0.0001] and wave III [noise intensity effect at 3 and 6 months for latency: F (1, 105) = 359.62, *p* < 0.0001 and F (1, 21) = 47.55, *p* < 0.0001; for amplitude: F (1, 140) = 47.72, *p* < 0.0001 and F (1, 42) = 16.76, *p* < 0.0001].

At 3 months, KO mice showed longer ABR latencies [genotype effect for wave I and II: F (1, 26) = 4.95 and 8.38, *p* = 0.032and 0.0076, respectively; Figure 2a,b] together with reduced ABR amplitudes [genotype effect for wave I and III: F (1, 28) = 5.1 and 7.72, *p* = 0.032 and 0.009, respectively; Figure 2d,f].

At 6 months, reduced ABR amplitudes were again detected in KO mice, but this time only for wave I [genotype effect: (F1, 18) = 11.75, *p* = 0.003; Figure 3d]. No difference between genotypes was found in the amplitude of the other waves [genotype effect for wave II and III: F (1, 15) = 2.33 and F (1, 4) = 1.95, ns; interaction genotype x sound intensity: F (1, 45) = 1.03 and F(1, 42) = 0.38, ns; Figure 3e,f], nor in the latencies [genotype effect for wave I, II and III: F (1, 7) = 0.001, F (1, 11) = 0.07 and F (1, 7) = 1.34, ns; interaction genotype x sound intensity: F (1, 28) = 0.565, F (1, 33) = 1.134 and F (1, 21) = 1.168, ns; Figure 3a–c].

### 2.2. Immunohistochemical Analysis in Inner Hair Cells (IHCs)

#### 2.2.1. RIBEYE Expression in IHCs

**Figure 4:**
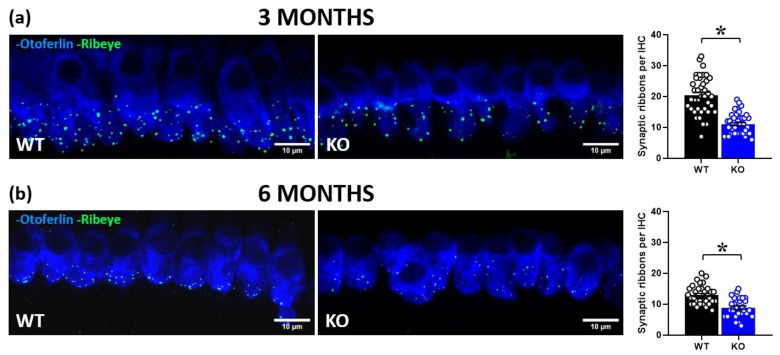
Quantitative analysis of synaptic ribbons in cochlear inner hair cells (IHCs). Immunofluorescent confocal imaging of ribbon synapses in cochlear IHCs at 3 and 6 months of age (**a**,**b**; **left panel**). Image resulted from stack reconstructions of ~20 slices of 0.3 μm thickness. Green indicates RIBEYE immunostaining of synaptic ribbons. Blue indicates otoferlin immunostaining, i.e., a specific marker of IHCs. Histograms (right side) represent the number of synaptic ribbons per IHC in the mid-turn of the cochlea at 3 and 6 months (**a**,**b**; **right panel**). Sample size: a total of 80 IHCs was analyzed for the 3-month-old group (obtained from 3 WT and 3 KO mice, ~13 cells/mouse) and a total of 59 IHCs for the 6-month-old group (obtained from 2 WT and 3 KO mice, ~10 cells/mouse). * *p* < 0.05. Data are mean ± SEM.

In order to gain insight into the peripheral mechanisms potentially underlying the acoustic alterations of mutant mice, we performed cochlear immunofluorescence analysis of RIBEYE, i.e., the major component of the synaptic ribbon that coordinates rapid and sustained vesicle release in IHCs (Figure 4). We observed a significant reduction in RIBEYE expression in KO mice compared to their WT littermates at 3 months of age [genotype effect: F (1, 78) = 74.71, *p* < 0.0001; Figure 4a]. This genotype difference was confirmed at 6 months [genotype effect: F (1, 57) = 25.34, *p* < 0.0001; Figure 4b], when an overall age-related reduction in the number of synaptic ribbons was detected, as expected [13].

#### 2.2.2. KCNMA1 Expression in IHCs

**Figure 5:**
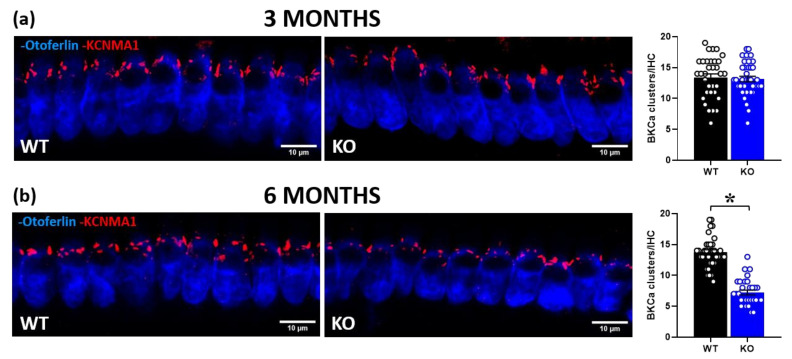
Quantitative analysis of BKCa channel expression in cochlear inner hair cells (IHCs). Immunofluorescent confocal imaging of BKCa channels in cochlear IHCs at 3 (**a**) and 6 months of age (**a**,**b**; **left panel**). Red indicates KCNMA1 immunostaining of the α subunit of BKCa channels, organized in dense clusters at the neck of the IHCs above nuclei. Blue indicates otoferlin immunostaining. Histograms represent the number of BKCa channel clusters per IHC in the mid-turn of the cochlea at 3 and 6 months (**a**,**b**; **right panel**). Sample size: a total of 70 IHCs were analyzed for the 3-month-old group (obtained from 3 WT and 3 KO mice, ~12 cells/mouse), and a total of 65 IHCs for the 6-month-old group (obtained from 2WT and 3 KO mice, ~10 cells/mouse). * *p* < 0.05. Data are mean ± SEM.

We then performed cochlear immunofluorescence analysis of BKCa channels in IHCs of 3- and 6-month-old mice (Figure 5). While at 3 months of age, BKCa expression did not significantly differ between genotypes [genotype effect: F (1, 68) = 0.1, ns; Figure 5a], at 6 months, the number of channel clusters was significantly reduced in KO mice compared to their WT littermates [genotype effect: F (1, 63) = 125.1, *p* < 0.0001; Figure 5b].

### 2.3. Therapeutic Effects of Chlorzoxazone (CHLOR) in Fmr1-KO Mice

**Figure 6:**
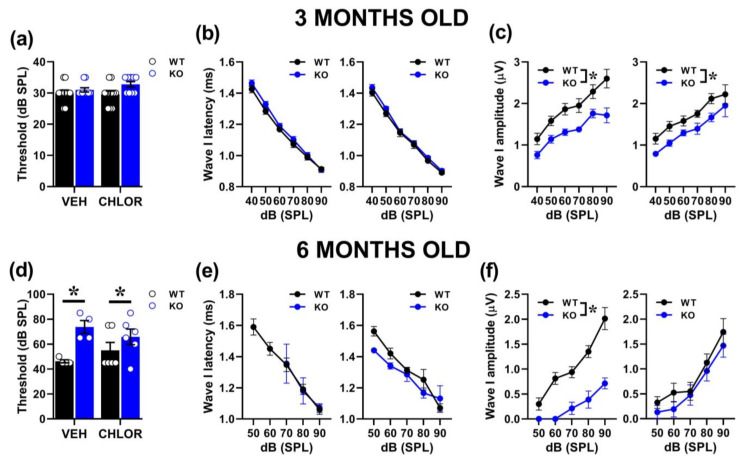
Acute effects of a BKCa channel agonist on the acoustic abnormalities of *Fmr1*-KO mice. The effects of a single administration of Chlorzoxazone (CHLOR; 5 mg/Kg, injected i.p. 1hr before testing) were assessed on ABR wave I parameters in 3 (**a**–**c**) and 6 (**d**–**f**) month-old mutant mice and their WT littermates. Sample size: for the 3-month-old group n = 11 (WT-VEH), 10 (KO-VEH), 11 (WT-CHLOR) and 9 (KO-CHLOR); for the 6-month-old group, n = 4 (WT-VEH and KO-VEH) and 6 (WT-CHLOR and KO-CHLOR). * *p* < 0.05. Representative examples of ABR traces are provided in Supplementary Figure S1. Data are mean ± SEM.

We evaluated the effects of CHLOR on ABRs focusing on wave I, since this ABR parameter was most markedly affected by genotype differences at both ages (Supplementary Figure S1). At 3 months, acute CHLOR did not rescue the reduced ABR amplitudes displayed by mutant mice [genotype effect: F (1, 37) = 23.72, *p* < 0.0001; treatment effect and its interaction with genotype: F (1, 37) = 0.87, F (1, 37) = 1.17, ns; Figure 6c]. As expected, based on our previous results, no differences between genotypes were detected on the thresholds [genotype effect: F (1, 37) = 3.88, treatment and its interactions, ns; Figure 6a] and the ABR latencies [genotype effect: F (1, 37) = 1.69, treatment and its interactions, ns; Figure 6b]. At 6 months of age, CHLOR rescued the reduced ABR amplitude displayed by mutant mice [interaction genotype x treatment: F (1, 16) = 3.99, *p* = 0.06; genotype effect from separate ANOVAs in VEH F (1, 6) = 43.82, *p* = 0.0006, in CHLOR: F (1, 10) = 0.81, *p* = 0.39; Figure 6f], but not their higher hearing thresholds [genotype effect: F (1, 16) = 10.417, *p* = 0.0053; interaction genotype x treatment: F (1, 16) = 1.969, ns; Figure 6d]. As expected, no genotype difference emerged on ABR latencies [genotype effect: F (1, 10) = 0.22, treatment and its interactions, ns; Figure 6e].

## 3. Discussion

Our findings provide, for the first time, an extensive characterization of the peripheral auditory phenotype of the *Fmr1*-KO mouse model of FXS at two adult ages, i.e., 3 and 6 months, representing the most common testing ages for adult mice. Mutant mice showed a hyperacusis-like phenotype, with increased startle responses, reduced ABRs, and reduced number of synaptic ribbons in cochlear IHCs. These alterations were observed at both ages and tended to be more pronounced and varied at 6 months, when a significant reduction in KCNMA1 expression in IHCs was also detected. In line with the selective BKCa deficit at this age, the acute administration of the BKCa agonist, CHLOR, was able to rescue the ABR alterations of *Fmr1*-KO mice at 6 months only. While the hyperacoustic phenotype of mutant lice was somehow expected, the age-specific differences in both the behavioral and BKCa profiles in cochlear IHCs represent the major novel finding of this study, together with its pharmacological approach.

Mutant mice of both ages showed an increased startle response, in line with previous preclinical results [21,22,30,31] and in agreement with the clinical FXS phenotype [7]. Interestingly, the hyperacusis-like profile of *Fmr1*-KO mice seemed more pronounced at 6 months, when an exaggerated startle response was evident at all noise intensities. A worsening with age was previously described for another common symptom of auditory hypersensitivity displayed by *Fmr1* mutant mice, i.e., their higher susceptibility to sound-induced seizures [38,46] and it was confirmed for other FXS-like phenotypes of this mouse model [23,24]. Hence, it is possible that an increase in the general behavioral impact of the lack of FMRP occurs with age in this mouse line, as already suggested [47]; further behavioral studies including multiple testing points at adulthood and during aging would be instrumental to further elucidate this issue. It is also possible that a more acoustic-specific effect of age modulated the impact of FMRP deletion. Here, we employed a startle-testing protocol based only on low intensity-sounds, since this is necessary to reveal a FXS-like hyperacusis profile in *Fmr1*-KO mice [21,22,30]. Interestingly, a similar startle hyperacusis phenotype specific to low sound intensities was associated with aging in B6 mice [13]. In these B6 aging mice, the paradoxical effect on startle and ABR responses was explained by a compensatory increased activity of the remaining/surviving IHC ribbon synapses. It could, therefore, be possible that *Fmr1*-KO mice experienced a sort of similar exaggerated or anticipated aging process of IHC ribbon synapses, explaining their increased startle responsiveness while their ABR waves were decreased.

The impression of an age-related exacerbation of the acoustic phenotype of mutant mice was somehow confirmed when the functional cochlear correlates of the startle response were evaluated. Three-month-old *Fmr1*-KO mice showed normal hearing thresholds compared to WT, for both click and tone bursts, and unaltered DPOAEs, indicating normal hearing and outer hair cells (OHCs) activity. Six-month-old mutant mice displayed instead increased hearing thresholds, especially at 8 kHz. At this age, both WT and KO animals showed low DPOAEs, confirming the early progressive hearing loss and related OHCs dysfunction displayed by B6 mice [36,37]. At both ages, a reduction in ABR wave-I amplitudes was observed in *Fmr1*-KO mice, especially at the highest noise intensities, in line with previous studies [32,33]. At 6 months, the ABR phenotype of mutant mice was indeed specific to wave I, in contrast to the genotype differences observed also on waves II and III in younger mice. Wave I is known to result from the synchronous activity of the auditory nerve fibers and reduction in its amplitude was correlated with loss of ribbon synapses [48]. Indeed, confocal immunofluorescence imaging of the synaptic ribbons in IHCs in the mid-frequency region of the cochlea (8–16 kHz) revealed a significant reduction in the number of ribbon synapses in *Fmr1* mutants at both ages. To our knowledge, this is the first evidence of a quantitative deficit in ribbon synapses in adult *Fmr1*-KO mice; this may suggest a major role of cochlear functional alterations in mediating the acoustic phenotypes of these mutants. Interestingly, the *Fmr1* mutant phenotype was observed against a background of a general reduction in synaptic ribbons with age, in agreement with previous results in aging WT mice [13].

While ribbon synaptic expression was altered in *Fmr1* mutants at both ages, BKCa channels were reduced in KO mice only at 6 months. The lack of deficits in the cochlear expression of KCNMA1 at 3 months of age was quite surprising, since deficits in this BKCa subunit were previously observed in several brain areas of *Fmr1*-KO mice around this age (Ref. [17]; nonetheless, a large age range was employed in this study, i.e., 3–5 months, and could still play a role in the discrepancy with our results). A strong link between the *Fmr1* mutation and BKCa functions was previously demonstrated. Direct interactions between FMRP and the KCNMA1 subunit of BKCa channels were documented by in vitro and in vivo studies [49,50,51], and the lack of FMRP led to reduced BKCa channel activity [17,50] and, therefore, neuronal hyper-excitability. Moreover, it was shown that BKCa channel activity is regulated by a broad spectrum of kinases, including CaMKII [52,53,54], which is a well-known target of FMRP [55].

The lack of deficits in KCNMA1 expression in 3-month-old mutant mice was a surprising result because of the well-known tight association between BKCa channel dysfunction and hyperacusis [56,57]. Our findings instead suggest that deficits in the IHC ribbon synapses, rather than BKCa, may be the initial primary factor underlying startle hyper-responsiveness and ABR deficits in *Fmr1*-KO mice. The secondarily late decrease in IHC BKCa expression at 6 months, associated with increased ABR deficits, underlines the progressive aspect of hearing loss in *Fmr1*-KO mice. It is, nonetheless, important to highlight that a deficit in KCNMA1 expression in IHCs is typically associated with aging in B6 mice [13]; hence, as previously discussed for the hyperacusis phenotype, the KCNMA1 expression profile of *Fmr1*-KO mice, characterized by a reduction at 6 months of age, may suggest an accelerated/anticipated aging process affecting the functionality of IHCs in these mutants [58]. It is worth recalling that BKCa channels are essential for fast IHC repolarization and precise timing of synaptic transmission at the auditory nerve fibers [59]. Future studies should investigate in depth the mechanisms underlying the link between BKCa function and hyperacusis in *Fmr1*-KO mice, an issue that we did not specifically address in our study. Additional measures of BKCa cochlear functionality, e.g., BK currents, in *Fmr1* mutants would be particularly useful to better understand the therapeutic potential of these channels to treat FXS. The age-specific efficacy of CHLOR demonstrated here should not represent a limitation to the therapeutic potential of this BKCa agonist to treat acoustic dysfunction in FXS. First, this age is among the most widely used in neurobehavioral research on mouse models of NDDs, including the *Fmr1*-KO line. For this specific mouse model, this adult age indeed corresponds to the most suitable to detect the most robust behavioral FXS-like phenotypes [24], as already mentioned before. Approximately at the same age (5 months), we previously observed therapeutic effects of CHLOR in *Fmr1*-KO mice on multiple behavioral alterations, including exaggerated startle responses [21]. Second, here, only the acute effects of CHLOR were evaluated; it is, therefore, still possible that a chronic activation of BKCa channels may induce compensatory effects on altered ribbon synapses and, in turn, be effective on ABR deficits also at 3 months of age.

A potential limitation of our study may lie in the lack of an additional age older than 6 months. This could have been interesting to assess the potential differences in aging processes in the acoustic phenotype of the *Fmr1* mouse model. To our knowledge, this issue has not been investigated yet and would definitely represent an interesting research domain. Nonetheless, the scope of our study was mainly related to the phenotype of *Fmr1*-KO mice at adulthood, since this is the most relevant for the study of this mouse model and the most interesting for therapeutic interventions. Furthermore, the investigation of the acoustic phenotype in mutants with the B6 background was complicated by the early hearing loss occurring in WT mice, thus rendering difficult the interpretation of the acoustic phenotypes at very advanced ages. Another possible limitation of our study was the exclusive focus on a single behavioral test, the acoustic startle response. Although this test is the most directly linked to hyperacusis and one of the most widely used in preclinical mouse models, future studies including additional measures of acoustic sensitivity and discrimination may be of interest to further support our results in *Fmr1*-KO mice.

In conclusion, our findings added to the characterization of the acoustic phenotype of the *Fmr1*-KO mouse model of FXS, highlighting the relevance of including multiple ages to assess a hyperacusis-like profile in this mutant line. These results warrant the efficacy of BKCa agonists to treat hyperacusis in FXS, which could be potentially extended to ASD and other NDDs. The use of Chlorzoxazone, i.e., a BKCa agonist already available on the market for non-developmental pathologies, enhances the clinical potential of our findings, supporting a re-purposing therapeutic approach. As with all preclinical research, caution is needed when extrapolating findings from mouse models to human patients. Further studies should aim to replicate these findings in human FXS populations and they would be critical to validate the potential therapeutic target and its relevance to auditory dysfunction in FXS.

## 4. Materials and Methods

### 4.1. Animals

Subjects were *Fmr1*-KO mutant mice and their wild-type littermates (maintained on B6 background). Only males were tested, as they are most commonly employed in neurobehavioral studies on FXS because of the higher prevalence of the pathology in the male sex [58] and the most common use of *Fmr1*-KO males in preclinical research [60].

Two cohorts of animals (i.e., 16 WT and 14 KO 3-month-old mice; 10 WT and 10 KO 6-month-old mice) were used for the entire study; they underwent the acoustic startle test, followed after one week by a first electrophysiological assessment of ABRs and DPOAEs. Mice were then retested after one week for their electrophysiological acoustic responses following a single i.p. injection of either a vehicle (VEH) or Chlorzoxazone (CHLOR) solution. The sample size for each dataset is further specified in each figure legend. The choice of the sample size was based on previous studies detecting significant genotype or treatment effects in the considered behavioral, electrophysiological, and histochemical measures. For the pharmacological study, data for 3-month-old mice were initially obtained from a subgroup of the cohort of behaviorally tested mice, consisting of 7 (WT-VEH), 6 (KO-VEH), 4 (WT-CHLOR), and 4 (KO-CHLOR). Since this first dataset led to significant genotype, but not treatment effects, we replicated the pharmacological experiments in 3-month-old mice in order to reach the following sample size (see also Figure 6): 11 (WT-VEH), 10 (KO-VEH), 11 (WT-CHLOR), and 9 (KO-CHLOR). All solutions were freshly prepared on each experimental day. CHLOR (Sigma Aldrich, Saint-Quentin-Fallavier, France) was dissolved in saline solution containing 1.25% DMSO (Sigma Aldrich, Saint-Quentin-Fallavier, France) and 1.25% Tween80 (Sigma Aldrich, Saint-Quentin-Fallavier, France). The same solution without drug was used for the VEH control group. The dose of 5 mg/kg was chosen based on previous studies, showing its efficacy on the acoustic and behavioral alterations of *Fmr1*-KO mice, without effects in WT littermates [21]. The day after the pharmacological test, mice were euthanized and cochlear samples were obtained from vehicle-treated mice of both genotypes.

All mice were bred in our animal facility at Bordeaux University. Breeding trios were formed by mating two *Fmr1* heterozygous (+/−) females with a C57BL/6J adult wild type male purchased from Janvier (Le Genest St. Isle, France). On post-natal day (PND), 3 pups were marked by paw tattoo, using a non-toxic odorless ink (Ketchum permanent Tattoo Inks green paste, Ketchum MFG. Co., New York, NY, USA), and tail samples were collected for DNA extraction and subsequent PCR genotype assessment [23]. Mice were weaned on PND 21 and housed in same-sex cages in groups of 3–5 animals/cage in polycarbonate standard cages (33 × 15 × 14 cm in size; Tecniplast, Limonest, France) and kept in an air-conditioned room (temperature 21 ± 1 °C; humidity 55%) with lights on from 07:00 a.m. to 07:00 p.m.

### 4.2. Acoustic Startle Response

Mice were assessed in the acoustic startle test in four startle chambers (SR-LAB, San Diego Instruments, San Diego, CA, USA). Each chamber consisted of a nonrestrictive cylindrical enclosure attached horizontally on a mobile platform, which was, in turn, resting on a solid base inside a sound-attenuated isolation cubicle. A high-frequency loudspeaker mounted directly above the animal enclosure inside each cubicle produced a continuous background noise and various acoustic stimuli in the form of white noise. Mechanical vibrations caused by the startle response of the mouse were converted into analog signals by a piezoelectric accelerometer attached to the platform. The sensitivity of the stabilimeter was routinely calibrated to ensure consistency between chambers and across sessions. Twenty-four hours before testing, mice were habituated to the testing chambers in the absence of any sound stimulus during a single 5 min session.

The protocol used in this study was previously employed in the *Fmr1*-KO mouse model [22,30]. It consisted of pulses of white sound of 20 ms duration and varying intensity: +6, +12, +18 and +24 dB over the 66dB background level (namely 72, 78, 84, and 90 dB). Each intensity was presented 8 times, in a randomized order with variable intervals (10 s to 20 s, mean = 15 s) between the onset of each pulse.

### 4.3. Auditory Brainstem Response (ABRs) and Distortion Product Otoacoustic Emissions (DPOAEs)

To record ABRs, each mouse was anesthetized with isoflurane and placed in a closed acoustic chamber, with its body temperature kept constant at 37 °C. For sound stimulus generation and data acquisition, we used a TDT RZ6/BioSigRZ system (Tucker-Davis Technologies, Alachua, FL, USA). Three subdermal electrodes were used: ground, reference, and active electrode placed at the tail, below the pinna of the left ear and at the forehead, respectively. Click-based ABR signals were averaged after the presentation of a series of 512 stimulations and sound intensities of 10–90 dB SPL in 10 dB step were tested. For frequency-specific ABRs, tone burst stimuli of 4 single frequencies (4, 8, 16 and 24 kHz) were presented. Threshold was determined as the lowest recognizable ABR response. The amplitude of ABR wave I was estimated by measuring the voltage difference between the positive (P1) and negative (N1) peak of wave I.

DPOAEs were tested by using two simultaneous continuous pure tones with a frequency ratio of 1.2 (*f*1 = 12.73 kHz and *f*2 = 15.26 kHz). DPOAEs were collected with the TDT RZ6/BioSigRZ system designed to measure the level of the “cubic difference tone” 2*f*1–*f*2 [13].

### 4.4. Tissue Preparation and Immunocytochemistry

Mice were deeply anesthetized with isofluorane (Vetflurane, Virbac, Suffolk, UK) and euthanized one day after the last electrophysiological assessment. Cochlear samples were fixed by incubation in 4% paraformaldehyde in phosphate-buffered saline (PBS), pH 7.4, at 4 °C overnight and washed with cold phosphate-buffered saline (PBS). They were then incubated overnight in PBS solution containing 10% EDTA pH 7.4 at 4 °C. The middle part of the organ of Corti (area encoding between 8 and 16 kHz) was then dissected and the tectorial membrane removed. The tissue was first incubated with PBS containing 30% horse and goat serum and triton X100 0.5% for 45 min at room temperature. IHCs were labeled with a mouse monoclonal antibody (Anti-Otoferlin1/400, AbCam, cat # ab53233). Synaptic ribbons were labeled with a guinea pig polyclonal antibody (Anti-RIBEYE 1/200, SYSY, cat # 192104). BKCa channels were labeled with a rabbit polyclonal antibody (Anti-KCNMA1 1/200, Alomone, Israel, cat # APC-021). Explants of Organ of Corti were incubated overnight at 4 °C with primary antibodies. The following fluorescent secondary antibodies 1/500 were then used: Fluoprobes 647H-donkey anti-Mouse IgG (Interchim, cat # FP-SC4110), Fluoprobes 488-donkey anti Guinea Pig IgG (Jackson, cat # 706-545-148), and Fluoprobes547H-donkey anti Rabbit IgG (Interchim, cat # FP-SB5110). For image acquisition, organ of Corti samples were analyzed using a confocal laser scanning microscope Leica DM6 CFS TCS SP8 with a 63× oil immersion objective (NA = 1.4) coupled with lasers 488 nm, 552 nm and 638 nm (at Bordeaux Imaging Center, Bordeaux, France). For complete 3D-stack reconstruction of IHCs, 25–30 images (0.3 μm thickness) were acquired. Numbers of synaptic ribbons and BKCa channels surfaces were calculated using the 3D-Objects Counter Plugin of ImageJ.

### 4.5. Statistical Analysis

Data from 3 and 6 months of age were separately analyzed, since they were independently collected. All data were analyzed by an ANOVA with genotype alone or combined with treatment as the between-subject factor and noise intensities as the within-subject factor, when appropriate. Post hoc pairwise comparisons (Fisher’s PLSD test, New York, NY, USA) were performed when a significant interaction was detected. All analyses were conducted using the software Statview (SAS institute, 5.0.1, Cary, NC, USA) and SPSS (PAWS Statistics 18, Chicago, IL, USA), and α was set at 0.05. Results are expressed as mean ± SEM throughout the text.

## Data Availability

The datasets used and analyzed during the current study are available from the corresponding author upon reasonable request.

## Supplementary Materials

The following supporting information can be downloaded at: https://www.mdpi.com/article/10.3390/ijms241411863/s1.
