# Supplementary material for: Hyperacusis in the Adult Fmr1-KO Mouse Model of Fragile X Syndrome: The Therapeutic Relevance of Cochlear Alterations and BKCa Channels
Source: Int J Mol Sci. 2023 Jul 24;24(14):11863. doi: 10.3390/ijms241411863 (PMC10380266; doi:10.3390/ijms241411863)
Supplement: Supplementary file 1 [file ijms-24-11863-s001.zip › ijms-2498329-supplementary.pdf]

International Journal of *Molecular Sciences*

MDPI

Article

Hyperacusis in the Adult Fmr1-KO Mouse Model of Fragile X Syndrome: The Therapeutic Relevance of Cochlear Alterations and BKCa Channels.

CelesteFerraguto[1], Yohan Bouleau [2,3], Thibault Peineau[2,3], Didier Dulon[2,3] and Susanna Pietropaolo[1,*]

[1] Univ. Bordeaux, CNRS, EPHE, INCIA, UMR 5287, F-33000 Bordeaux, France.
[2] Neurophysiologie de la Synapse Auditive, INSERM UA06, Université de Bordeaux, Bordeaux, France.
[3] Institut de l'Audition, Centre Institut Pasteur/Inserm UA06, Paris, France.
* Correspondence: susanna.pietropaolo@u-bordeaux.fr

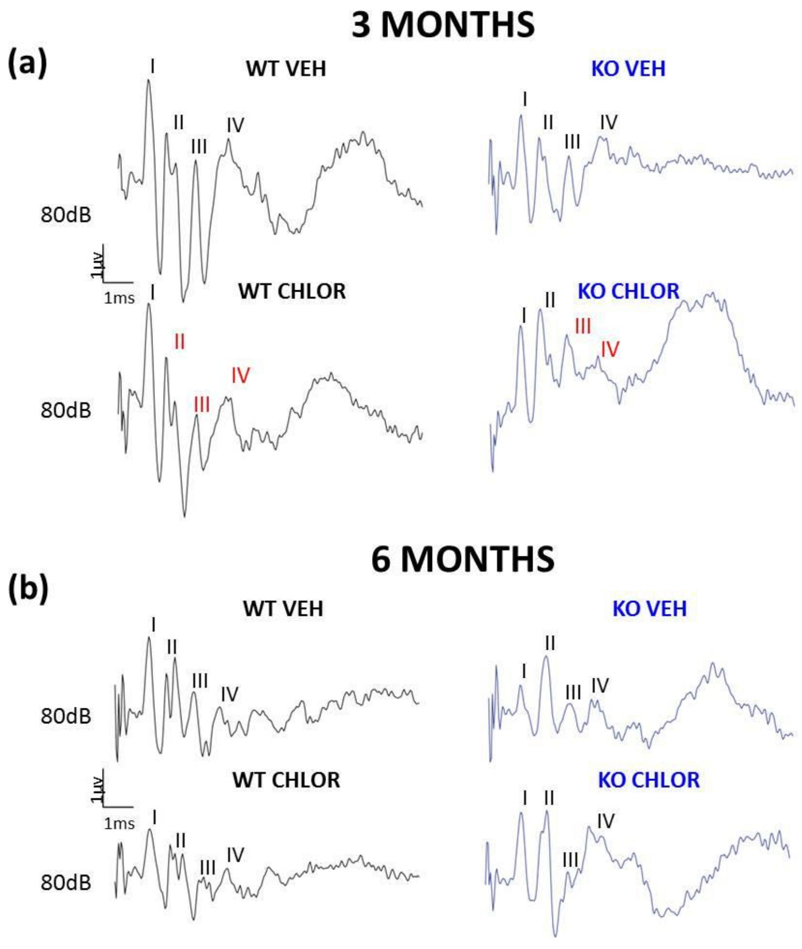

Figure S1: Representative examples of ABR traces, evoked by clicks of 80 dB SPL in 3-month-old (a) and 6-month-old (b) WT and KO mice, acutely treated with vehicle (VEH) or 5 mg/Kg chlorzoxazone (CHLOR).
